# *Etlingera elatior*-Mediated Synthesis of Gold Nanoparticles and Their Application as Electrochemical Current Enhancer

**DOI:** 10.3390/molecules24173141

**Published:** 2019-08-29

**Authors:** Farah Asilah Azri, Jinap Selamat, Rashidah Sukor, Nor Azah Yusof, Nurul Hanun Ahmad Raston, Noordiana Nordin, Nuzul Noorahya Jambari

**Affiliations:** 1 Laboratory of Food Safety and Food Integrity, Institute of Tropical Agriculture and Food Security, Universiti Putra Malaysia, 43400 UPM Serdang, Selangor, Malaysia; 2 Department of Food Science, Faculty of Food Science and Technology, Universiti Putra Malaysia, 43400 UPM Serdang, Selangor, Malaysia; 3 Department of Chemistry, Faculty of Science, Universiti Putra Malaysia, 43400 UPM Serdang, Selangor, Malaysia; 4 School of Bioscience and Biotechnology, Faculty of Science and Technology, Universiti Kebangsaan Malaysia, 43600 UKM Bangi, Selangor, Malaysia

**Keywords:** gold nanoparticles, green synthesis, *Etlingera elatior*, torch ginger, electrochemical

## Abstract

This work presents a simple green synthesis of gold nanoparticles (AuNPs) by using an aqueous extract of *Etlingera elatior* (torch ginger). The metabolites present in *E. elatior*, including sugars, proteins, polyphenols, and flavonoids, were known to play important roles in reducing metal ions and supporting the subsequent stability of nanoparticles. The present work aimed to investigate the ability of the *E. elatior* extract to synthesise AuNPs via the reduction of gold (III) chloride hydrate and characterise the properties of the nanoparticles produced. The antioxidant properties of the *E. elatior* extract were evaluated by analysing the total phenolic and total flavonoid contents. To ascertain the formation of AuNPs, the synthesised particles were characterised using the ultraviolet-visible (UV-Vis) spectroscopy, Fourier transform infrared (FTIR) spectroscopy, high-resolution transmission electron microscopy (HRTEM), energy-dispersive X-ray (EDX) microscopy, and dynamic light scattering (DLS) measurement. The properties of the green synthesised AuNPs were shown to be comparable to the AuNPs produced using a conventional reducing agent, sodium citrate. The UV-Vis measured the surface plasmon resonance of the AuNPs, and a band centered at 529 nm was obtained. The FTIR results proved that the extract contained the O-H functional group that is responsible for capping the nanoparticles. The HRTEM images showed that the green synthesized AuNPs were of various shapes and the average of the nanoparticles’ hydrodynamic diameter was 31.5 ± 0.5 nm. Meanwhile, the zeta potential of −32.0 ± 0.4 mV indicates the high stability and negative charge of the AuNPs. We further successfully demonstrated that using the green synthesised AuNPs as the nanocomposite to modify the working surface of screen-printed carbon electrode (SPCE/Cs/AuNPs) enhanced the rate of electron transfer and provided a sensitive platform for the detection of Cu(II) ions.

## 1. Introduction

The application of metallic nanoparticles is gaining traction in various fields due to their unique electrochemical and optical properties. These unique properties make them an ideal nanomaterial to be used in biosensor applications. The electrochemical properties of AuNPs can enhance the electrode conductivity by facilitating electron transfer, and thus improving the sensitivity. Metallic nanoparticles of explicit sizes and morphologies can be readily synthesised by means of chemical and physical methods [1]. The AuNPs provide advantages in many applications including biomedical, drug delivery, photothermal therapy, tissue or tumour imaging, labelling and sensing [2,3].

Increasing awareness towards green chemistry and other biological processes in recent years has led the need to develop an eco-friendly approach for the synthesis of AuNPs. This is largely due to the relative ease of the production, good control of sizes and shapes, unique optical and electronic properties, and good biocompatibility of the AuNPs. Green synthesis or plant-mediated biological synthesis of nanoparticles is drawing attention from the scientific community due to its cost-effectiveness, simplicity, ease of up-scaling, and safety. Green synthesis is a bottom-up approach where the nanoparticles are built from smaller entities and then assembled to produce the final particles [4]. The AuNPs synthesis requires a protective or stabilising agent to adsorb onto the surface of the newly formed nanoparticles to prevent particles agglomeration and further growth. The most commonly used stabilising agents reported are sodium citrate (Na_3_C_6_H_5_O_7_), transferrin, sodium borohydride (NaBH_4_), and cetyltrimethylammonium bromide (C_19_H_42_BrN), which are known as the chemical method that can subsequently produce toxic wastes [5]. Therefore, alternative non-toxic reagents are often sought after to improve the biocompatibility of AuNPs.

Several biological agents such as bacteria, fungi, algae and plants have been applied in the green synthesis of metallic nanoparticles. However, the use of plant materials for the synthesis of AuNPs has been demonstrated to be more advantageous than microbiological-based methods as (1) it eliminates the elaborate process of maintaining the bacterial and fungal cultures [6], (2) the nanoparticles synthesised by plant extract are shown previously to be more stable, and (3) the production rate is faster than those synthesised using microorganisms [7]. Plant extracts act as reducing agents as well as stabilising agents, which are believed to greatly influence the characteristics of the nanoparticles [8]. The reduction process of metal ions during the formation of nanoparticles is affected by several factors, such as the nature of plant extracts that contains active biomolecules at different combinations and concentrations, pH of the reaction mixture, incubation temperature, reaction time, and electrochemical potential of the metal ions [9]. Plant biomolecules, which are a large group of polyphenolic compounds that comprise of anthocyanins, isoflavonoids, flavonols, chalcones, falvones, and flavanones, have been shown to actively chelate and reduce metal ions into nanoparticles [10,11]. The protective and reductive activities of this plant biomolecules are accountable for the reduction of gold ions.

There have been a number of reports on the synthesis of various shapes of AuNPs using extracts from fruits, stalks, or leaves of different plants such as Ocimum sanctum (holy basil) [12], Nepenthes khasiana (pitcher plant) [13], Azadirachta indica (neem) [14], Basella alba (vine spinach) [15], Allium sativum (garlic) [16], Ficus benghalensis (banyan) [17], and Carica papaya (papaya) [18] but with different efficacies. *Etlingera elatior* or commonly known as torch ginger is a fast-growing plant that is widely cultivated and abundantly available throughout Southeast Asia. The inflorescence *E. elatior* is a fleshy pink flower that grows at the tip of stalks with bracts spreading. The *E. elatior* flowers have immense ornamental values and these young flowering shoots are commonly added to various dishes especially in Malaysia, Indonesia, and Singapore to improve the taste and smell. Previous studies on the characterisation of the chemical composition of *E. elatior* extracts by GC-MS revealed more than 30 major organic compounds, which includes fatty alcohols (Z-11-Pentadecenol, dodecanol, 1-Hexadecanol), fatty acids (dodecanoic acid, tetradecanoic acid), fatty aldehydes (dodecanal, 1-Tetradecyl acetate), tannins, flavonoids, saponins, and steroids [19,20,21]. In addition to the antioxidative, anticancer, and antimicrobial properties of these organic compounds [19,20,22], the abundance of these compounds in *E. elatior* makes the plant a promising source of reducing and stabilising agents for the green synthesis of AuNPs.

In this study, we aimed to support and contribute additional evidence on the ability of *E. elatior* as a reductant and stabiliser in the AuNP synthesis [12], and also explore the potential application of the *E.elatior*-mediated synthesised AuNPs as an electrode modifier. The resulting modified electrode was further applied as an electrochemical metal ion detection system to measure copper ions by differential pulse stripping voltammetry in the aqueous solution.

## 2. Results and Discussion

### 2.1. Determination of Phenolic and Flavonoids Compounds

Different plant sources contain different concentrations and combinations of reducing agents that may affect the characteristics of the nanoparticles [8]. The reduction of the metal ions was attributed to the phenolics, terpenoids, polysaccharides and flavones compounds present in the extract [9]. Total phenolic compounds (TPC) in plants are known to act as free radical scavengers and it is believed that the antioxidant activity of the most plant produce is mostly due to the presence of phenolic compounds [13]. The antioxidative mechanism of polyphenolic compounds depends on their hydrogen donating and metal ion chelating capabilities [14,15]. Gallic acid (GA) was used as the standard for the calibration curve and TPC was expressed as the gallic acid equivalent (GAE) in mg per 100 g of the sample. The calibration equation for gallic acid was;

y = 0.0112x + 0.0253 (R^2^ = 0.999) (1)

where y is the absorbance and x is the GA concentration in mg/mL. In this study, the TPC concentration of the *E. elatior* extract was 193.5 ± 1.03 mg GAE/100 g sample.

We then determined the flavonoids content of the *E. elatior* extract based on the aluminium chloride colourimetric assay. The calibration equation for quercetin was;

y = 0.0024x + 0.0113 (R^2^ = 0.996) (2)

where y is the absorbance and x is the QE concentration in mg/mL. The total flavonoids content (TFC) of the *E. elatior* extract was 135.5 ± 1.34 mg QE/100 g sample. Flavonoids are the most common and extensively distributed single group of phenols found in plants [16]. Flavanoids, as highly effective antioxidants, can chelate metal ions with their carbonyl groups or π-electrons and avert metal-initiated lipid oxidation by forming complexes with metal ions [15]. These compounds are believed to be involved during the initiation of nanoparticles nucleation and further aggregation or bioreduction stage [10]. The tautomeric conversions of flavonoids from the enol-form to the keto-form may release a volatile hydrogen atom that can reduce metal ions to form nanoparticles [10].

The type of extraction solvent used has been shown to greatly influence the amount of antioxidants extracted from the *E. elatior* flower [17]. Sepahpour et al. [17] and Wijekoon et al. [18] reported that water was the least effective solvent for the phenolic extraction from *E. elatior* compared to other organic solvents used, such as methanol, ethanol and acetone. Table 1 shows the amount of TPC and TFC extracted using different percentages of methanol. Based on the results, 50% of methanol in the extraction solvent produced higher TPC while 100% of methanol produced more the TFC content. However, in this study, we used 10% of methanol as the extraction solvent to minimise any detrimental effect during the AuNPs synthesis procedure. We discovered that the amount of phenolics and flavonoids extracted from the fresh *E. elatior* using water with 10% of methanol was sufficient to be used as the reducing and stabilising agent for the AuNPs synthesis.

### 2.2. Characterisation of Synthesised Gold Nanoparticles

#### 2.2.1. Characterisation via Physical Observation and Ultraviolet-Visible Spectroscopy

**Figure 1:**
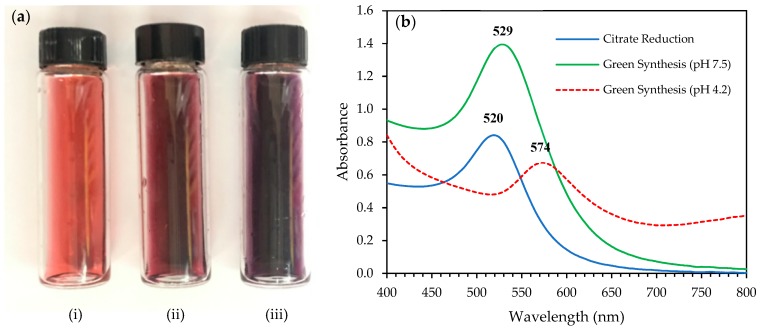
(**a**) Gold nanoparticles (AuNPs) solutions; i. red wine colour of AuNPs solution synthesised by the citrate reduction method, ii. deep red colour of AuNPs solution synthesised by the green synthesis method (pH 7.5), and iii. purple colour of AuNPs solution synthesised by the green synthesis method (pH 4.2). (**b**) UV-visible spectrum of synthesised AuNPs by respective methods with the scale of wavelength between 400 and 800 nm.

A rapid colour change of gold solution from light yellow to deep red in the reaction mixture was observed shortly after the addition of the *E. elatior* extract. The complete reduction of gold ions was observed within 15 min. The colour exhibited by metallic nanoparticles was due to the coherent excitation of all free electrons within the conduction band, leading to an in-phase oscillation known as localised surface plasmon resonance (LSPR) with a specific wavelength of incident light [19]. The LSPR of AuNPs produced a strong absorbance band in the visible region that could be measured by the UV-Vis spectroscopy. The colour change indicates the different sizes and shapes of AuNPs due to the excitation of surface plasmon vibrations. Figure 1a shows the differences in the colour of the AuNPs solutions. The colour intensity of the AuNPs solution synthesised using the *E. elatior* extract was higher than to the AuNPs solution synthesised via the citrate reduction method.

Our initial attempt to synthesise AuNPs using a mildly acidic pristine extract of *E. elatior* resulted in the formation of a dark purple solution. Further LSPR confirmation of the synthesised AuNPs by the UV-vis spectroscopic analysis showed that the SPR peak for the green synthesised AuNPs using the pristine *E. elatior* extract was observed at 574 nm (Figure 1b). Here we assumed that the nanoparticles produced were in a large size and tend to aggregate. This is because the aggregation of AuNPs subsequently resulted in the shift of the absorption band to longer wavelength due to the electric dipole-dipole interaction and coupling between the plasmons of neighbouring particles in the form of aggregates [20].

However, by adjusting the pH of the *E. elatior* extract from 4.2 to 7.5, a deep red solution was produced and a shift in the SPR wavelength to 529 nm was observed. pH was shown to play a significant role in controlling the size and morphology of the produced nanoparticles [21,22]. Singh and Srivastava [23] reported that there was a gradual shift in the absorption on the synthesis of AuNPs using the black cardamom extract when pH was increased from highly acidic to neutral. This wavelength shift indicates that the particle size was reduced, and vice versa when the pH was increased from neutral to highly alkaline. There was a slight red shift in the SPR wavelength at 529 nm for the *E. elatior*-mediated AuNPs compared to the peak of citrate-capped AuNPs at 520 nm. However, the sharp and symmetrical SPR peaks were indicative of small spherical nanoparticles. Based on the absorbance profile, the higher absorbance peak of the green synthesised AuNPs compared to the citrate-capped AuNPs suggests that a higher concentration of the AuNPs were produced.

#### 2.2.2. Characterisation via Fourier Transform Infrared Spectroscopy

**Figure 2:**
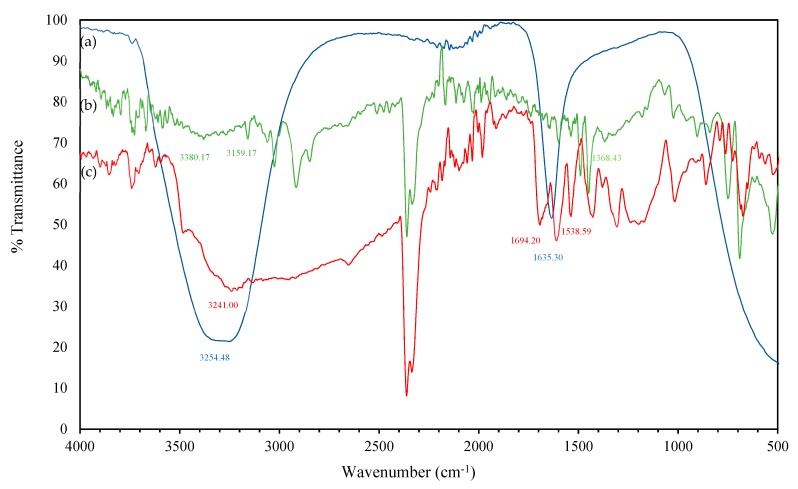
FTIR spectrum of (**a**) aqueous extract of *E. elatior*; (**b**) purified AuNPs synthesised by the green synthesis method using the *E. elatior* extract, and (**c**) purified AuNPs synthesised by the citrate reduction method.

The functional groups of the compounds responsible for the formation and stabilization of AuNPs were identified using FTIR spectroscopy. The AuNPs that were synthesised using plant extracts were shown to be coated with a thin layer of organic material that acted as capping agents [24]. Green synthesis causes the nanoparticles to be surrounded by the plant metabolites including sugars, terpenoids, polyphenols, alkaloids, phenolic acids and proteins. These plant metabolites play important roles in the reduction of metal ions into nanoparticles and supporting their subsequent stability [10]. The FTIR main signals are indexed in Figure 2 while the details on the IR bands were provided in the supplementary materials (Table S1). The FTIR spectrum of the *E. elatior* extract in Figure 2a shows the band at 3254 and 1635 cm^−1^. The intense broad absorbance at 3254 cm^−1^ is the characteristic of the hydroxyl (stretch O-H bonded, strong broad) functional group in alcohols and phenolic compounds. The highest absorption peak at 3254 cm^−1^ reflected the -OH group that were potentially responsible for the reducing properties of the *E. elatior* extract [25]. Meanwhile, the band at 1635 cm^−1^ could be amide I band of the proteins released by the *E. elatior* or C=C groups/aromatic rings [26]. All these peaks reveal the presence of highly concentrated alcohol or phenol, aromatic amines and amide (I) of proteins in the extract.

The FTIR spectrum of the AuNPs synthesised by the green synthesis method (Figure 2b) shows bands at 1368, 3159, and 3380 cm^−1^ along with other small bands. The decrease in the peak intensity observed after the encapsulation of chloroaurate ion suggest the possible contribution of the mentioned groups. The band at 1368 cm^−1^ that corresponds to the C-N stretching vibration of aliphatic amines or alcohols/phenols may be responsible for the reduction and capping of AuNPs. Meanwhile, the band at 3380 and 3159 cm^−1^ correspond to the amide II bands (N-H) of proteins. The N-H vibrational bands become weaker and broader in the spectrum of stabilised AuNPs. This indicates that the AuNPs synthesised using the *E. elatior* extract were surrounded by some proteins and metabolites. Figure 2c represents the FTIR spectrum of AuNPs synthesised by the citrate reduction method, which shows the band at 3241, 1694, and 1538 cm^−1^ along with other small bands. The band at 3241 cm^−1^ represents strong and broad O-H stretching which refer to the intermolecular bonds of the molecules in sodium citrate (Na_3_C_6_H_5_O_7_) that has three -OH groups in the structure. A peak present at 1694 cm^−1^ denotes the C=N stretching of imine/oxime compounds. Oximes are usually generated by the reaction of hydroxylamine with aldehydes or ketones. The peak at 1538 cm^−1^ is due to strong N-O stretching from the nitro compound that was also present in sodium citrate.

All three FTIR spectra (Figure 2a–c) clearly show the changes in the -COOH group for -OH, i.e., the hydroxyl group at the peak of 3254 cm^−1^ which appeared in the plant extract. However, the peak was narrower and shifted to 3159 and 3241 cm^−1^ following the encapsulation of nanoparticles. These FTIR results support the finding in Section 2.1, which suggested that the *E. elatior* extract is rich in phenolics with sufficient amount hydroxyl and carboxyl groups. These hydroxyl and carboxyl groups possess oxidation-reduction abilities to bind metals and inactive them from chelation. Therefore, it can be suggested that the hydroxyl groups are the main contributor in the reduction of Au (III) to Au (0) through the oxidation of hydroxyl to the carbonyl group, which can be presented as:

AuCl_4_^−^ + 3R-OH → Au^0^ + 3R-O + 3H^+^ + 4Cl^−^ (3)

#### 2.2.3. Characterisation via High-Resolution Transmission Electron Microscopy and Energy-Dispersive X-ray

**Figure 3:**
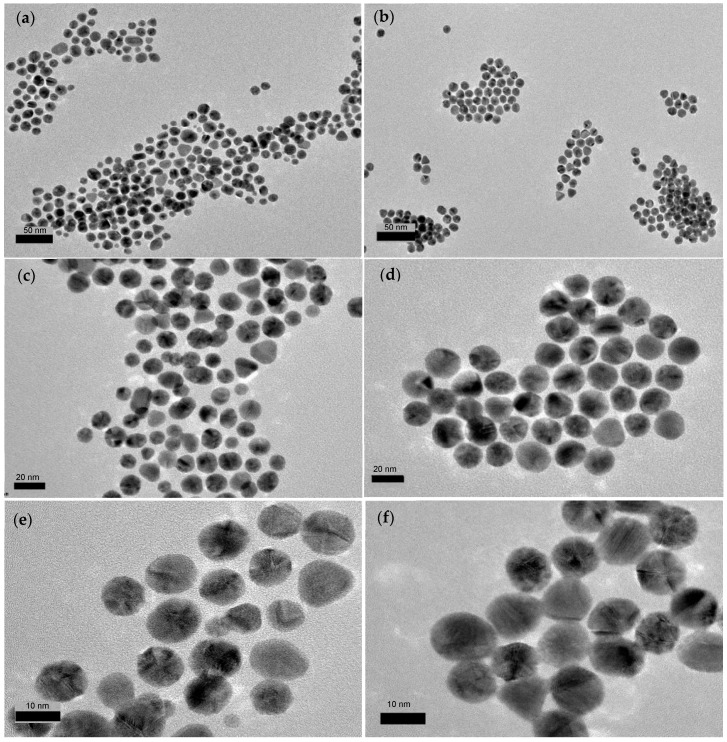
High-Resolution Transmission Electron Microscopy (HRTEM) images of the *E. elatior-mediated* AuNPs at (**a**) 50,000X magnification; (**c**) 100,000X magnification; (**e**) 250,000X magnification and in comparison, with AuNPs synthesised by sodium citrate at (**b**) 50,000X magnification; (**d**) 100,000X magnification; (**f**) 250,000X magnification.

The HRTEM images in Figure 3 display clear shapes of the AuNPs which confirm their formation. The newly formed AuNPs using the *E. elatior* extract observed were polydisperse and consisted of a mixture of spherical, triangular and rod-shaped with irregular contour particles (Figure 3a,c,e). The triangular and rod-shaped particles formed (Figure S3) were shown to have high surface areas while various sizes of spherical nanoparticles were observed. The nanoparticles formed were in the range of 3–25 nm in size, as the diameter measured manually. The morphologies of the green synthesised AuNPs were then compared to the AuNPs synthesised using the standard citrate reduction method (Figure 3b,d,f). The citrate-capped AuNPs was shown to be more uniformly spherical in shape (Figure 3d) and present in almost similar size. Both green and citrate synthesised AuNPs were shown to be stable with no further aggregation and colour changes when the solutions were stored for up to three months at 4 °C. The possible nucleation and growth for different shapes of AuNPs is influenced by different components present in the plant extract. Based on the study by Jiang et al. [27] reducing sugars might be responsible for the formation of spherical particles which also prevents the nanoparticles from aggregation. Meanwhile, flavones correspond to the fast nucleation and growth of the flower-like structures with the help of steric hindrance of ketones [27]. Well-defined triangular and hexagonal shaped AuNPs were believed to be synthesised by the polyphenol molecules of the plant extract. The reacted adjacent phenolic hydroxyls of polyphenols are inductively oxidized to the corresponding carbonyls and ketones due to the oxidation-reduction potential of Au(III).

**Figure 4:**
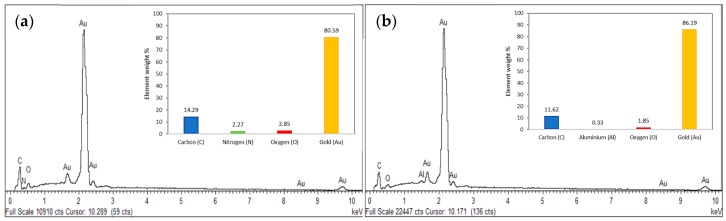
Energy Dispersive X-ray (EDX) spectrum showing the presence of gold elements and other bioorganic components in (**a**) AuNPs synthesised by *E. elatior*; (**b**) AuNPs synthesised by sodium citrate. Insert graphs represent the elements distribution percentage in the samples.

The EDX spectrum profiles in Figure 4 gives a clear indication that other elements were involved in the synthesis of the AuNPs. This analysis confirmed that the gold was the highest elementary composition. The detection of strong gold (Au) signal along with weak signals of carbon (C), nitrogen (N) and oxygen (O) suggests the presence of biomolecules bound to the surface of the AuNPs from the capping with the organic materials of the *E. elatior* extract (Figure 4a). This infers the presence of stabilising molecules on the green synthesised AuNPs. The EDX spectrum of AuNPs synthesised via the citrate reduction method (Figure 4b) shows that there is aluminium (Al) present as impurities in the sample coming from the sample substrate but no N was present. Based on the EDX spectrum analysis, the proposed green synthesis method has successfully produced 80.6% of gold as compared to 86.2% by the citrate reduction method, which demonstrates its great potential as an alternative method to synthesis AuNPs.

#### 2.2.4. Characterisation via Dynamic Light Scattering (DLS)

The DLS measurement is known to measure the shell thickness of a capping or stabilizing agent enveloping the synthesised nanoparticles along with the actual size of the metallic core [20]. DLS reveals the hydrodynamic size of the particles and not only determines the average size but also the size distribution. The Gaussian distribution data is provided in the supplementary material (Figure S4). The data revealed a bimodal distribution and the highest peak with the larger fraction was observed at particle size of 37.4 nm with 83.7% intensity. The smaller fraction reveals the larger size of nanoparticles at 416.1 nm with 16.3%, which proved the presence of aggregated particles in the sample. Moreover, the large particle size might also be because of the various forces of interaction in the solution including van der Waals forces [20]. This was also supported by the previous HRTEM images where the variety of size and shape of particles could be clearly observed. Based on the DLS measurements, the z-average size of the green synthesised AuNPs and citrate reduction was 31.5 ± 0.5 nm and 20.6 ± 0.4 nm, respectively. The particles size measured by DLS was larger compared to the HRTEM results. This is due to the circumstance that the measured size also comprises the bio-organic compounds enveloping the core of the AuNPs. However, the results confirmed that the green synthesis method using *E. elatior* is comparable to the conventional chemical method, in terms of producing small (< 50 nm) AuNPs. Meanwhile, surface charge is commonly quantified by the zeta potential which delivers information on the net charge of particles in a liquid condition. The value of the zeta potential is closely related to the suspension stability and particle surface coating [28]. The zeta potential of the green AuNPs was −32.0 ± 0.4 mV, compared to −57 ± 0.2 mV of the citrate AuNPs. These negative zeta potentials confirmed the formation of negative charges on the surface of the AuNPs.

### 2.3. Electrochemical Properties of the Modified SPCE

**Figure 5:**
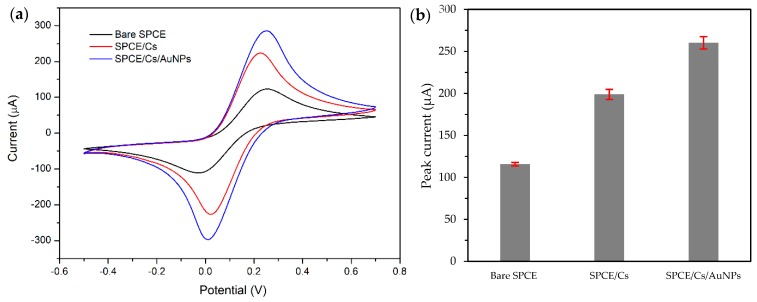
(**a**) Cyclic voltammograms and (**b**) bar graph of bare screen-printed carbon electrode (SPCE), SPCE and chitosan (SPCE/Cs) and SPCE with a mixture of AuNPs and chitosan (SPCE/Cs/AuNPs) (green synthesised) modified electrodes in 5 mM [Fe(CN)6]^3−/4−^ in phosphate buffer saline (PBS).

In this study, we further applied the green synthesised AuNPs as the screen-printed carbon electrode (SPCE) surface modifier by incorporating chitosan (Cs) as the polymer matrix. SPCE emerges as alternatives to the conventional electrodes due to their suitability for the on-site applications. Meanwhile, chitosan is a non-toxic polymer that offer biocompatibility, high mechanical strength, good adhesion on electrochemical surfaces, and subsequently enhance the binding of the negatively charge AuNPs [29]. Based on Figure 5, the peak current of the SPCE modified with a mixture of AuNPs and chitosan (SPCE/Cs/AuNPs) increased by 153.95 µA relative to bare SPCE and was 26% higher compared to SPCE/Cs (213.31 µA). These peak increments could explain the enhanced electrocatalytic activity and larger surface area of AuNPs as it can improve the mass-transport rate of electrons between the SPCE and [Fe(CN)6]^3−/4−^ ions, and therefore inducing faster electron transfer kinetics. Furthermore, the larger electrochemical surface-active area is not only beneficial for enhancing the sensor sensitivity, but also advantageous for aiding the electron transfer [30]. Most of the reported works on green synthesis gold nanoparticles were applied as antibacterial and antifungal. However, Bastos-Arrieta et al. [31] synthesised silver nanoparticles (AgNPs) using grape stalk waste extract for modification of screen-printed electrodes and proved their suitability for sensing purposes. Meanwhile, Emmanuel et al. [32] fabricated the glassy carbon electrode with the AuNPs synthesised by *Acacia nilotica* twig bark extract and proved that the electrode exhibited outstanding recovery results and an excellent reduction ability compared to the unmodified electrode.

### 2.4. Detection of Copper (Cu) Ions using SPCE/Cs/AuNPs

**Figure 6:**
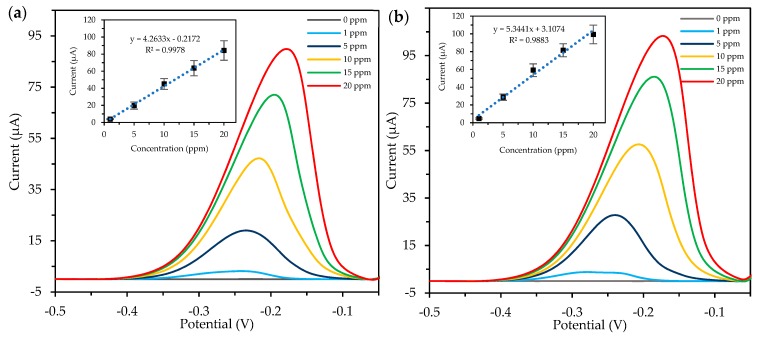
Detection of Cu^2+^ ions in free solution by the differential pulse voltammetry analysis in 0.1M KCl/HCl buffer. (**a**) SPCE modified with chitosan and green synthesised AuNPs and (**b**) SPCE modified with chitosan and citrate-capped AuNPs.

The comparison of the analytical performance of the SPCE modified with citrate-capped AuNPs and green AuNPs was determined based on the detection of Cu^2+^ ions in the solution. The detection and was carried out by the differential pulse voltammetry (DPV) analysis in six increasing concentrations of Cu(II) ranging from 0 to 20 ppm. In principle, the detection of Cu^2+^ ions using the adsorptive stripping voltammetry (ASV) method involves three main steps including adsorptive accumulation, electrochemical reduction of a surface-active complex of the metal and stripping out [33]. The signal of the surface-confined species is correlated to the surface concentration, through the adsorption isotherm, providing the relationship between the surface and bulk concentrations of the adsorbate [34]. Hence, to evaluate the efficiency of the modified SPCE, a control experiment was performed using the bare SPCE (without any modification) and the result is provided in the supplementary material (Figure S5). Based on the result, the calibration plot of Cu^2+^ detection by the bare SPCE displays nonlinearity at lower concentrations which support that the electrochemical transduction material including AuNPs as an electrode modifier contributes to the electrochemical enhancement. Meanwhile, Figure 6 shows the DPV responses of both modified electrodes and the calibration plots were presented as an insert. Well-defined peaks for Cu^2+^ detection by SPCE modified with green AuNPs and citrate-capped AuNPs can be observed at between −0.24 to −0.17 V and −0.27 to −0.17 V, respectively. The peaks currents were proportionally increased with positive shifts of peak potentials as the concentration of Cu(II) increase. The DPV peak currents were obtained with correction to the base line and the calibration plots were evaluated from the peak currents. The results proved that the SPCE modified with green synthesised AuNPs performed well as the electrode modifier and subsequently enhanced the sensitivity of the electrode. Although the current produced by SPCE modified with citrate-capped AuNPs were slightly higher due to smaller size of nanoparticles compared to green synthesised AuNPs, however, the linearity and stability of green synthesised AuNPs were observed to be greater.

## 3. Materials and Methods

### 3.1. Materials

**Figure 7:**
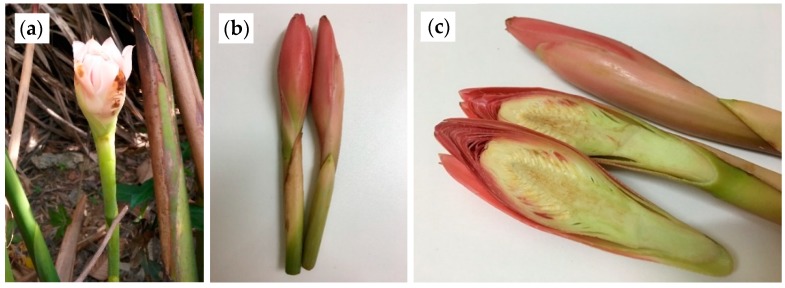
(**a**) Half-bloom inflorescence of *E. elatior*, (**b**) young shoots of *E. elatior* and (**c**) cross-section of the *E. elatior* flower.

Gold (III) chloride hydrate (HAuCl_4_.xH_2_O) was purchased from Sigma Aldrich (St. Louis, MO, USA). All other reagents including methanol (MeOH), sodium chloride (NaCl), Folin-Ciocaltae (FC) reagent, sodium carbonate (Na_2_CO_3_), sodium nitrite (NaNO_2_), aluminium chloride (AlCl_3_), sodium hydroxide (NaOH), gallic acid (QAE), quercetin (QE), potassium chloride (KCl), hydrochloric acid (HCl), nitric acid (HNO_3_), trisodium citrate (Na_3_C_6_H_5_O_7_), potassium ferrocyanide (K_4_Fe(CN)_6_), potassium ferricyanide (K_3_Fe(CN)_6_), copper (II) chloride (Cu) and phosphate buffer saline (PBS) tablet were of analytical grade with maximum purity. All aqueous solutions and suspensions were prepared using autoclaved purified water (Milli-Q RG, Millipore, Germany). The fresh *E. elatior* (Figure 7) was purchased from the wet market in Sri Serdang, Selangor, Malaysia, and was immediately extracted upon purchase.

### 3.2. Preparation of Etlingera elatior Extract

The *E. elatior* flowers were washed thoroughly and chopped. Approximately, 20 g of the chopped flower was boiled for 1 h in 100 mL of deionised water with the addition of 10 mL of methanol (to initiate the isolation of bioactive compounds). The extract was then filtered using filter paper (125 mm Ø, Whatman, Maidstone, United Kingdom), and the pH was adjusted to 7.5 with 1 M of NaOH. The extract solution was stored at 4 °C until further use.

### 3.3. Determination of Polyphenolic Compounds of Etlingera elatior Extract

#### 3.3.1. Total Phenolic Content (TPC)

The concentration of total phenolic content was estimated by the Folin-Ciocalteu method with minor modifications [35]. Two hundred microlitres of the *E. elatior* extract was mixed with 1000 µL of the FC reagent (dilution ratio of FC reagent, 1:10 v/v) and left in the dark for 6 min. After that, 800 µL of 7.5% Na_2_CO_3_ solution were added, shaken and left in the dark for 2 h to react. The absorbance was recorded at 740 nm, and the total phenolic content of the *E. elatior* extract was expressed as the gallic acid equivalent (GAE/100 g) of the *E. elatior* flower. The standard curve was constructed using a standard solution of gallic acid (Figure S1).

#### 3.3.2. Total Flavonoid Content (TFC)

The total of flavonoids content in the *E. elatior* extract was measured using the spectrophotometric method as described by Kamboj et al. [36]. Briefly, 1 mL of the *E. elatior* extract was mixed with 0.3 mL of 5% NaNO_2_ and left for 5 min before added with 0.3 mL of 10% AlCl_3_. The mixture was mixed and 6 min later 2 mL of 1M NaOH was added to neutralize the solution. The absorbance of all samples was recorded at 510 nm using the UV-Vis spectrophotometer. Total flavonoids content was expressed in a mg quercetin equivalent (QE/100 g) of the *E. elatior* flower. The standard curve was constructed using a standard solution of quercetin (Figure S2).

### 3.4. Green Synthesis of Gold Nanoparticles using the E. elatior Extract

Aqua regia was prepared to clean the flask and magnetic stirring bar prior to the AuNPs synthesis. The procedure was conducted inside the fume hood with proper ventilation due to the strong acid odour. A 250-mL conical flask with a magnetic stirrer was placed in a container with ice cubes. Concentrated HCl and HNO_3_ were poured into the flask at a ratio of 3:1. The orange-red solution was formed, and the solution was allowed to settle for approximately 45 min. Following this, the aqua regia solution was carefully discarded, and the flask and magnetic stirrer bar were rinsed with distilled water multiple times and dried. Next, 34 mg of the HAuCl_4_ powder was measured in an Eppendorf tube, and 2 mL of sterile deionised water was added. The yellow solution was filtered using the dual-filter syringe nylon filter (13 mm Ø, 0.45 µm pore size, Thermo-Line, New South Wales, Australia), and 1.6 mL filtrate was added into 80 mL of sterile deionised water. The gold solution was poured into the cleaned flask and heated to 250 °C until bubbles formed while stirring at 70 rpm. Next, 8 mL of the *E. elatior* extract was added quickly to the boiled solution, and the stirring speed was increased to 200 rpm. The heating and stirring were continued for another 15 min. For the cooling step, the solution was kept stirred at 120 rpm without heating until it reached room temperature. The flask was covered with aluminium foil and kept at 4 °C until further use. The conventional citrate-capped AuNPs were also synthesised using the same method by replacing the green reducing agent of the *E. elatior* extract with 38.8 mM of Na_3_C_6_H_5_O_7_ solution.

### 3.5. Characterisation of Synthesised Gold Nanoparticles

#### 3.5.1. Ultraviolet-Visible Spectroscopy

UV-Vis spectroscopy was used to determine the formation and stability of AuNPs in the aqueous solution. The nanoparticle solution was diluted (1:2 dilution) with deionised water to minimise errors due to the high optical density of the solution. Next, 2 mL of the aliquot was measured in the UV-Vis spectra (Genesys 10S UV-Vis Spectrophotometer, Thermo Scientific, Waltham, MA, USA) using a 10-mm quartz cuvette. The scanning was done at a resolution of 1 nm between 200 to 900 nm with a fast mode scanning speed.

#### 3.5.2. Fourier Transform Infrared Spectroscopy

The AuNP dried samples were prepared by centrifuging the synthesised solution at 15000× *g* for 15 min at 25 °C. The solid residual layer was dispersed in sterile deionised water thrice to remove residual biological impurities. The pure residue was then oven-dried overnight at 40 °C. The obtained powder was subjected to the FTIR spectrometer (Nicolet 6700, Thermo Scientific, Waltham, MA, USA) measurement at a resolution of 4 cm^−1^.

#### 3.5.3. High-Resolution Transmission Electron Microscopy and Energy-Dispersive X-ray

The surface morphology of the AuNPs was analysed using HRTEM (JEM-2100F, JEOL, Tokyo, Japan). The samples were prepared by drying a droplet of the synthesised AuNPs suspension on a copper grid (Formvar Carbon Film, FCF400-CU, 400 Mesh, EMS, Hatfield, PA, USA), overnight at room temperature. The excessed solution was removed using filter paper. Energy-Dispersive X-ray was done using the field emission scanning electron microscopy, FESEM (UHR SU8030, Hitachi, Tokyo, Japan).

#### 3.5.4. Dynamic Light Scattering (DLS) Measurements

The size and stability of the AuNPs were measured using a Zeta-sizer instrument (Malvern Nano-ZS, Malvern Panalytical, Worcestershire, United Kingdom). The measurement of the zeta potential is based on the direction and velocity of particles under the influence of the known electric field. The material refractive index was set to *n* = 0.20, and material absorption, *k* = 3.32, using water as the dispersant.

### 3.6. Modification of Screen-Printed Carbon Electrode (SPCE) using Synthesised AuNPs

#### 3.6.1. Preparation of Modified SPCE

The AuNPs solution was used as it is after the synthesis process without any purification step or dilution. The screen-printed carbon electrode (SPCE) (Biogenes, Selangor, Malaysia) was drop-coated using the nanocomposite of AuNPs and chitosan (Cs). First, 0.5% Cs solution was prepared in 1% acetic acid and the mixture was stirred until fully dissolved. The nanocomposite was prepared by mixing 100 µL of 0.5% Cs solution with 100 µL of AuNPs solution (1:1, v/v). Then, 10 µL of the nanocomposite mixture was drop-coated onto the working electrode of SPCE. The SPCE was dried at room temperature overnight.

#### 3.6.2. Electrochemical Characterisation of Modified SPCE

The electrochemical performance of the AuNPs was tested using the cyclic voltammetry (CV) analysis. The electrochemical properties of bare SPCE were compared to the electrochemical properties of the AuNPs modified SPCE. The measurement was done in 5 mM of [Fe(CN)6]^3−/4−^ redox pair prepared in 10 mM phosphate buffer saline (PBS). The CV potential was set from −0.5 to 0.7 V with the scan rate of 0.05 V/s. The CV measurement was done using the Potentiostat/Galvanostat µStat 400 (Metrohm DropSens, Llanera, Asturias, Spain).

#### 3.6.3. Detection of Heavy Metal using Modified SPCE

The electrochemical performance of the modified SPCE was evaluated by detection of Cu(II) in 0.1 M KCl/HCl buffer at room temperature. The measurement was done using the differential pulse voltammetry (DPV) analysis with 300 s of deposition time at −1.2 V deposition potential, 0.005 V potential step, 0.025 V amplitude and 20 Hz frequency. The experiment was done in triplicate (n = 3).

## 4. Conclusions

In the present work, we reported a simple and novel method to synthesise AuNPs via the reduction of aqueous HAuCl_4_ ions using the *E. elatior* extract. The developed method resulted in the formation of AuNPs with spherical, triangular and some rod shapes with an average size of 31.5 ± 0.5 nm. The spectroscopic characterisation of the green synthesised AuNPs by UV-Vis, FTIR, HRTEM and EDX analyses further confirmed the formation and stability of the synthesised AuNPs. The overall results showed that this green synthesis method is comparable to the standard citrate-based method and can be considered as an alternative method. The synthesised AuNPs was further used as the electrode surface modifier which has been shown to greatly enhance the electrochemical current by enhancing the rate of electron transfer. The modified SPCE was tested for the detection of Cu(II), providing good sensitivity and linearity. The green synthesis method we proposed herein is eco-friendly, economical and can be considered for use in a large-scale production of the AuNPs by employing the abundantly available *E. elatior* plant.

## Figures and Tables

**Table 1:** Total phenolic and total flavonoid content in *the E. elatior* extract extracted using different percentages of methanol in the extraction solvent.

Extraction solvent	TPC (mg GAE/100 g)	TFC (mg QE/100 g)	References
100% water	90.7 ± 1.7	182.5 ± 3.2	[18]
100% methanol	361.2 ± 17.1	762.8 ± 44.5	[18]
90% methanol	431.4 ± 25.8	632.6 ± 51.2	[18]
50% methanol	615 ± 14.6	717.6 ± 41.0	[18]
10% methanol	193.5 ± 1.0	135.5 ± 1.3	This study

## Supplementary Materials

Figures S1–S5 and Table S1 are available in the supplementary materials.
