# Supplementary material for: Etlingera elatior-Mediated Synthesis of Gold Nanoparticles and Their Application as Electrochemical Current Enhancer
Source: Molecules. 2019 Aug 29;24(17):3141. doi: 10.3390/molecules24173141 (PMC6749185; doi:10.3390/molecules24173141)

Supplementary materials

Etlingera elatior-Mediated Synthesis of Gold Nanoparticles and Their Application as Electrochemical Current Enhancer

Farah Asilah Azri [1,*], Jinap Selamat [1,2,*] Rashidah Sukor [1,2], Nor Azah Yusof [3], Nurul Hanun Ahmad Raston [4], Noordiana Nordin [1] and Nuzul Noorahya Jambari [1,2]

Figure S1: Standard curve of gallic acid

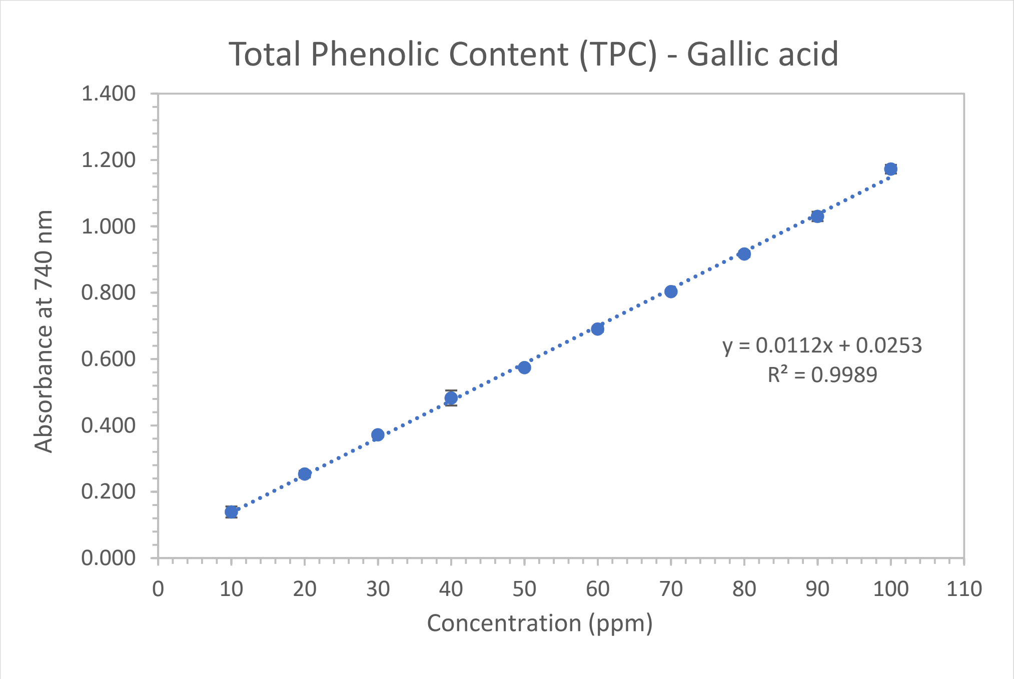

Figure S2: Standard curve of quercetin

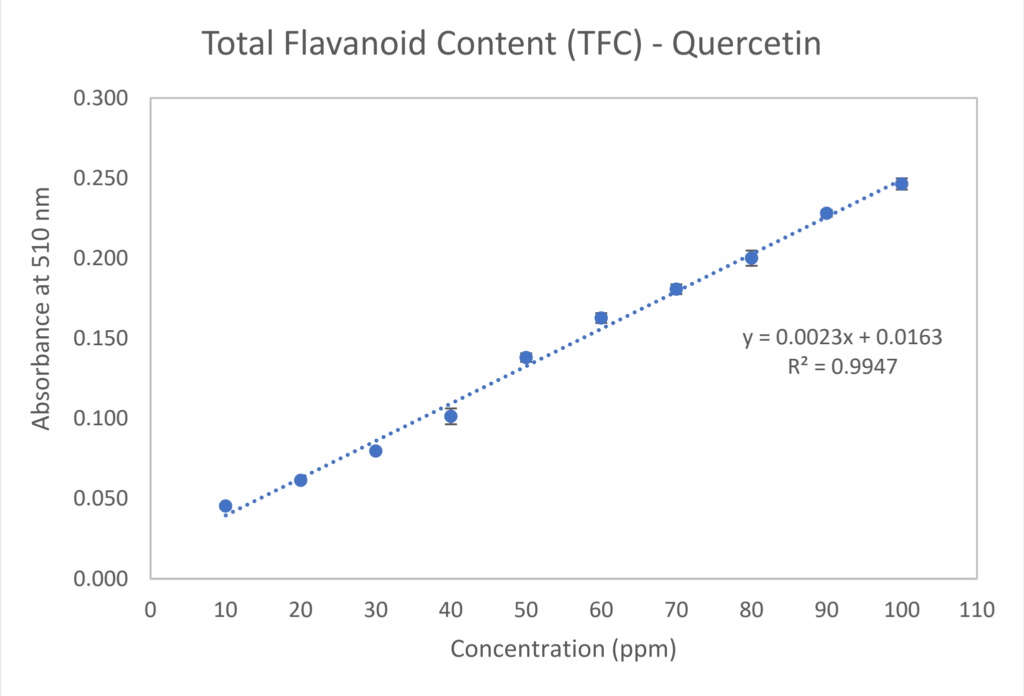

Table S1: Comparison of the FTIR bands present in gold nanoparticles synthesised by citrate reduction and green synthesis method.

Peaks Absorption (cm^{-1})	Citrate Reduction	Functional group	Green synthesis	Functional group
3000 – 3700	3741	-	3727	-
	3481	O-H (Alcohol), C-H (Alkene), N-H (Amide)	3670	O-H (Alcohol), C-H (Alkene)
	3241	O-H (Alcohol), O-H (Acid), N-H (Amide)	3380	O-H (Alcohol), N-H (Amide)
	3137	O-H (Acid), N-H (Amide)	3159	O-H (Acid), N-H (Amide)
		-	3025	O-H (Acid), C-H (Aromatic)
2000 – 2999	2653	O-H (Acid)	2916	C-H (Alkene), O-H (Acid)
	2485	-	2847	O-H (Acid)
	2361	-	2360	-
	2335	-	2333	-
1000 – 1999	1694	C=O (Carbonyl)	1599	-
	1611	-	1489	-
	1538	N-O (Nitro)	1448	-C-H (Alkane)
	1429	-C-H (Alkane)	1368	-C-H (Alkane), C-F (Alkyl Halide), C-N (Amine), N-O (Nitro)
	1379	-C-H (Alkane), C-F (Alkyl Halide)	1073	O-H (Alcohol), C-F (Alkyl Halide), C-O (Ether), C-O (Ester)
	1307	C-F (Alkyl Halide), C-O (Acid)	1034	C-F (Alkyl Halide), C-O (Ether), C-O (Ester)
	1197	C-F (Alkyl Halide), C-N (Amine), C-O (Ether), C-O (Ester)	-	
	1016	C-F (Alkyl Halide), C-O (Ether), C-O (Ester)	-	
500 - 999	860	=C-H (Alkene)	965	=C-H (Alkene)
	673	C-Cl (Alkyl Halide)	904	=C-H (Alkene)
	530	C-Br (Alkyl Halide)	847	=C-H (Alkene)
			749	=C-H (Alkene)
			690	C-H (Alkyl Halide), =C-H (Alkene)
			526	C-Br (Alkyl Halide)

Figure S3: The triangular and rod-shaped particles (circled in red) observed in green synthesised AuNPs.

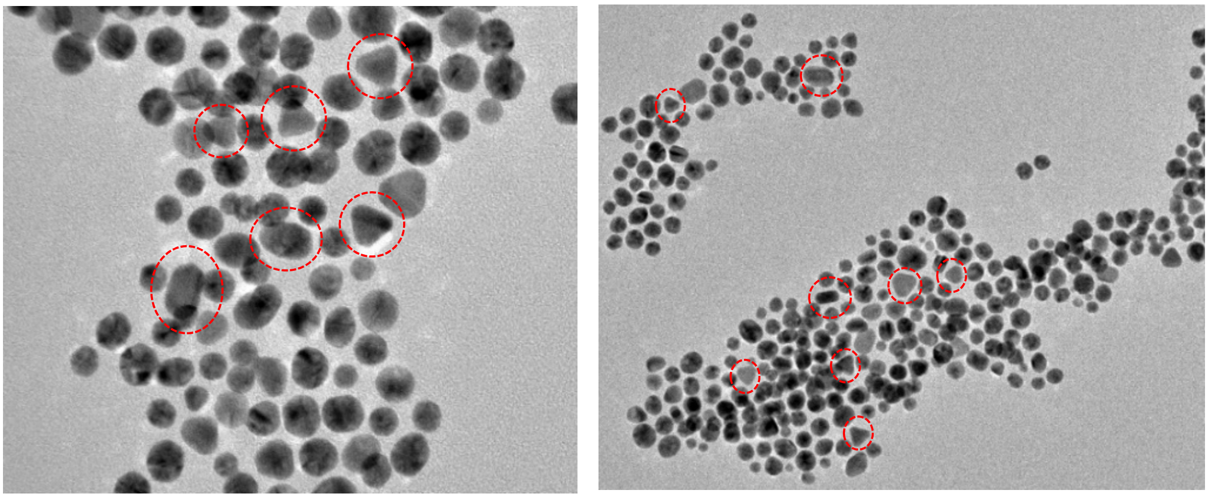

Figure S4: The Gaussian distribution data of DLS measurement of the green-synthesised AuNPs.

			Size (d.nm):	% Intensity:	St Dev (d.n...
Z-Average (d.nm):	31.14	Peak 1:	37.38	83.7	24.25
PdI:	0.401	Peak 2:	416.1	16.3	253.4
Intercept:	0.900	Peak 3:	0.000	0.0	0.000
Result quality :	Good				

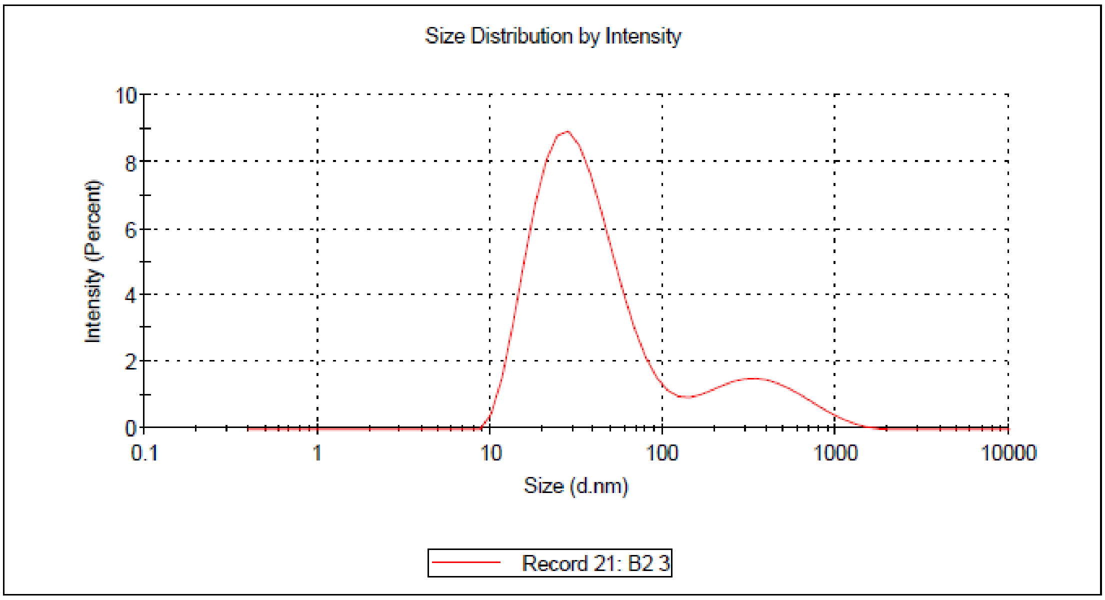

Figure S5: Detection of Cu^{2+} ions using bare SPCE in free solution by differential pulse voltammetry analysis. The measurements were done in 0.1M KCl/HCl buffer.

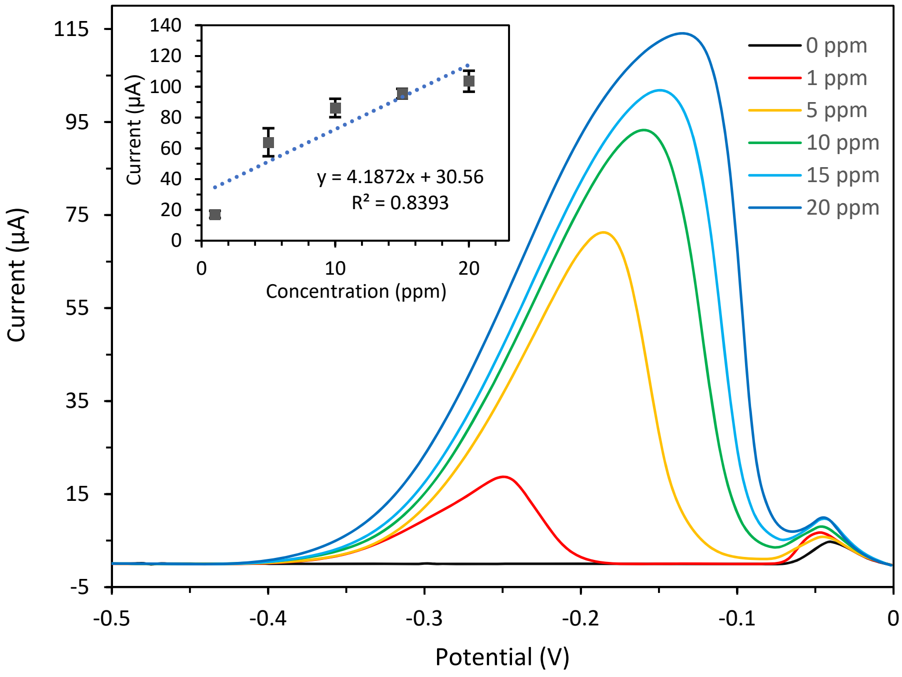

Supplement: Supplementary file 1 [file molecules-24-03141-s001.pdf]
